# A New Neuroanesthetic Protocol of Rendering Specialized Care in Treating Degenerative Lumbar Spine Diseases in High-Risk Patients: Prospective Analysis of the Results

**DOI:** 10.17691/stm2024.16.3.06

**Published:** 2024-06-28

**Authors:** A.A. Kalinin, V.Yu. Goloborodko, Yu.Ya. Pestryakov, R.A. Kundubayev, M.Yu. Biryuchkov, A.V. Shchegolev, V.A. Byvaltsev

**Affiliations:** MD, PhD, Associate Professor, Doctoral Candidate, Department of Neurosurgery and Innovative Medicine; Irkutsk State Medical University, 1 Krasnogo Vosstaniya St., Irkutsk, 664003, Russia; Neurosurgeon, Center of Neurosurgery; Russian Railways–Medicine Clinical Hospital, 10 Botkin St., Irkutsk, 664005, Russia; PhD Student, Department of Neurosurgery and Innovative Medicine; Irkutsk State Medical University, 1 Krasnogo Vosstaniya St., Irkutsk, 664003, Russia; Head of the Department of Anesthesiology and Resuscitation No.1; Russian Railways–Medicine Clinical Hospital, 10 Botkin St., Irkutsk, 664005, Russia; MD, PhD, Doctoral Candidate, Department of Neurosurgery and Innovative Medicine; Irkutsk State Medical University, 1 Krasnogo Vosstaniya St., Irkutsk, 664003, Russia; Assistant, Department of Neurosurgery with the Course of Traumatology; West Kazakhstan Marat Ospanov Medical University, 68 Maresyev St., Aktobe, 030019, Kazakhstan; MD, DSc, Professor, Head of the Department of Neurosurgery with the Course of Traumatology; West Kazakhstan Marat Ospanov Medical University, 68 Maresyev St., Aktobe, 030019, Kazakhstan; MD, DSc, Professor, Head of the Department of Military Anesthesiology and Resuscitation; S.M. Kirov Military Medical Academy, 6 Academician Lebedev St., Saint Petersburg, 194044, Russia; MD, DSc, Professor, Head of the Department of Neurosurgery and Innovative Medicine; Irkutsk State Medical University, 1 Krasnogo Vosstaniya St., Irkutsk, 664003, Russia; Chief of the Center of Neurosurgery; Russian Railways–Medicine Clinical Hospital, 10 Botkin St., Irkutsk, 664005, Russia; Professor, Department of Traumatology, Orthopedics and Neurosurgery; Irkutsk State Medical Academy of Postgraduate Education, 100 Yubileyny Microdistrict, Irkutsk, 664049, Russia

**Keywords:** lumbar spine, degenerative disease, decompressive-stabilizing interventions, minimally invasive spine surgery, enhanced postoperative recovery, clinical decision support system

## Abstract

**The aim of the study** is to assess the effectiveness of a new neuroanesthetic protocol for treating degenerative lumbar spine diseases in high-risk patients.

**Materials and Methods:**

Two groups of patients with a high risk of anesthesia and surgery determined by the authors’ clinical decision support system (CDSS) have been prospectively studied. A new neuroanesthetic protocol was used in the experimental group (EG, n=25), while the control group (CG, n=25) underwent intravenous anesthesia based on propofol and fentanyl. Minimally invasive transforaminal lumbar interbody fusion was performed in all cases. Changes of the intraoperative mean arterial pressure and heart rate, intensity of the local pain syndrome, amount of the opiates used, presence of cognitive disorders, adverse effects of anesthesia, and surgical complications have been compared.

**Results:**

The groups were representative (p>0.05) in terms of the age-gender parameters, anthropological data, comorbid background, involvement in smoking, preoperative characteristics of the lumbar spine, as well as the level of cognitive functions. No statistically significant changes of the mean arterial pressure (p=0.17) were registered in EG patients relative to the CG (p=0.0008). Intraoperative reduction of the heart rate in patients of the CG was not noted (p=0.49) in comparison with the EG (p=0.03). In the postoperative period, the best indicators of cognitive functions on the FAB test (p=0.02) and MoCA test (p=0.03) were revealed in EG. A significantly less amount of perioperative opiates (p=0.005) at a low level of the local pain syndrome was also noted (p=0.01). The intergroup analysis has shown fewer adverse effects of anesthesia in EG compared to CG (p=0.01) with a comparable number of postoperative surgical complications (p=0.42).

**Conclusion:**

A new neuroanesthetic protocol of rendering a specialized care to patients with a high risk of anesthesia and surgery, assessed by the authors-developed CDSS, has resulted in effective elimination of the local postoperative pain syndrome, reduction of perioperative application of opioids, and stabilization of intraoperative indicators of cardiovascular activity. In addition, no postoperative cognitive disorders, anesthetic side-effects, adverse pharmacological consequences of the complex usage of non-steroidal anti-inflammatory drugs, prolonged local anesthetics, alpha-2-agonist, and non-narcotic analgesics have been registered.

## Introduction

It has been presently established that increase of life expectancy in the population is connected with the increased number of surgical interventions in spinal diseases [1]. Various puncture, decompressive, and decompressive-stabilizing techniques are usually used in operative vertebrology [2]. The most common treatment of patients with significant degenerative changes in the supporting elements and impaired spatial interactions of vertebral segments is rigid fixation of the spine through the anterior, lateral and posterior approaches [3]. These operations, directed to decompression of neural structures, elimination of pathological mobility, and restoration of the sagittal spine alignment, are followed by a local postoperative pain syndrome of varying degrees of intensity [4].

Opioid analgesics are considered generally accepted for effective anesthesia after surgical intervention connected with direct injury of the soft tissues [5]. Ineffective pain management after the operation has been established to be associated with a longer period of treatment and recovery [6]. At the same time, longterm application of opiates may result in drug addiction, intestine dysfunction, and urinal retention [7].

The results of spinal interventions depend on a number of factors: the character of the operation and method of anesthesia [8, 9], constitutional features, patient’s physical status, and comorbidities [10, 11]. In this connection, special attention is presently payed to the improvement of postoperative outcomes in patients with high neuroanesthetic risks: elderly age, obesity, comorbidities, or their combinations [12, 13]. This cohort of patients needs a multidisciplinary tactics [14], which includes an individual choice of anesthesia method and type of neurosurgical intervention as well as prophylaxis of possible complications with preventive correction of the existing general somatic risks [15].

Objectification and justification of choosing individual neuroanesthetic approach are based on the doctor’s clinical experience and clinical decision support systems (CDSS) [16].

Lack of data on the application of a complex operative anesthetic tactics for treating patients with high risks of postoperative complications and neuroanesthetic risks has determined the relevance of this study.

**The aim of the study** is to assess the effectiveness of a new neuroanesthetic protocol for treating degenerative lumbar spine diseases in high-risk patients.

## Materials and Methods

We have studied the results of treating patients operated on the lumbar spine using minimally invasive decompressive-stabilizing techniques at the Russian Railways–Medicine Clinical Hospital in the Center of Neurosurgery (Irkutsk, Russia) in the period from February to August 2022. According to the authors’ CDSS [17], all patients had a high risk of anesthesia and surgery (over 8 points). The prospective pilot observational study was carried out in compliance with the Declaration of Helsinki (2013) and has been approved by the Ethical Committee of Irkutsk State Medical University (protocol No.1 of February 24, 2021).

Patients with repeated operations on the lumbar spine; competitive diseases on the lumbar level (infectious inflammatory pathology, traumatic injury, tumors); osteoporosis; drug allergic reactions in the history; dementia prior to the operation: the score below 16 on the Montreal Cognitive Assessment (MoCA) test and below 12 on the Frontal Assessment Battery (FAB) cognitive test, and those who refused to participate in the study were excluded from the investigation.

Taking into consideration the anesthesia methods, patients were divided into two groups (n=25 in each): control (CG) and experimental (EG). All patients were administered intravenous anesthesia with rocuronium bromide, propofol, and fentanyl in the standard dosages and supported by artificial lung ventilation. A new neuroanestheic protocol [18] was additionally used in patients of the EG. It included preoperative intramuscular introduction of ketoprofen (100 mg), infiltration of soft tissues with 0.75% ropivacaine (10 ml) before the incision, intraoperative intravenous introduction of dexmedetomidine (0.2–0.4 μg/kg/h), postoperative intramuscular introduction of paracetamol (1000 mg). During the operation, the depth of narcosis and neuromuscular conductivity were assessed according to the conventional methodology.

In all cases, minimally invasive surgical technologies were used: transforaminal lumbar interbody fusion through paramedian intermuscular approaches and percutaneous transpedicular stabilization. The anesthetic protocols were alternated until 25 patients were recruited in each group

To compare the groups, the following indicators have been analyzed:

intraoperative characteristics and parameters of the postoperative period (anesthesia duration; the amount of the opiates used; parameters of intraoperative hemodynamics: heart rate, mean arterial pressure; the need in postoperative analgesia expressed in OME (oral morphine equivalents); duration of staying in the post anesthesia care unit (PACU); length of hospital treatment);

intensity of postoperative pain syndrome according to visual analogue scale (VAS) during hospitalization period;

cognitive functions before surgical intervention and on day 5 after it assessed by MoCA and FAB tests;

the number of adverse effects of anesthesia (by clinical data);

the number of surgical complications.

**Statistical analysis** was performed using Statistica for Windows 13.5 software (StatSoft Inc., USA). As there were no normal distribution according to Shapiro–Wilk, Kolmogorov–Smirnov, and Lilliefors tests, methods of nonparametric statistics were applied. The results were presented by median, values of the first and third quartiles (Me [Q1; Q3]). The Mann–Whitney, Wilcoxon tests and χ^2^ criterion were employed for the comparison analysis. Differences were considered statistically significant at p<0.05.

## Results

The description of the groups is presented in Table 1. The comparison intergroup analysis did not reveal any statistically significant differences in gender, age, constitutional features, physical status defined by the American Society of Anesthesiologists (ASA) classification system, comorbid diseases, smoking status, character of the pathology, number of operated segments, and preoperative level of cognitive functions.

**T a b l e 1:** General characteristic of patients in the studied groups

Parameters	Experimental group (n=25)	Control group (n=25)	p
Age (years), Me [Q1; Q3]	64 [60; 72]	65 [59; 74]	0.72
Males/females, n (%)	17(68)/8(32)	20(80)/5(20)	0.36
BMI, Me [Q1; Q3]	27.5 [24.9; 29.1]	26.9 [24.4; 28.8]	0.54
ASA risk, grade, n (%):			0.47
II	4(16)	4(16)	
III	15(60)	16(64)	
IV	6(24)	5(20)	
Comorbidities, n (%):			0.15
diabetes mellitus	7(28)	5(20)	
hypertension	8(32)	9(36)	
lung pathology	5(20)	2(8)	
kidney pathology	2(8)	5(20)	
gastrointestinal diseases	3(12)	4(16)	
Smoking, n (%)	6(24)	4(16)	0.23
Pathology, n (%):			0.81
herniated disk with segmental instability	9(36)	6(24)	
spinal stenosis	4(16)	2(8)	
spondylolisthesis	10(40)	13(52)	
local kyphotic deformity	2(8)	4(16)	
Number of operated segments, n (%):			0.08
1	4(16)	6(24)	
2	13(52)	12(48)	
3	8(32)	7(28)	
Cognitive functions before operation (score), Me [Q1; Q3]:			
MoCA	27.5 [27.0; 29.0]	28.0 [27.0; 30.0]	0.12
FAB	17.5 [16.0; 18.0]	17.0 [16.0; 18.0]	0.94

The intergroup comparison has found similar parameters of anesthesia duration and length of hospital stay (p=0.27 and p=0.06, respectively) (Table 2). The duration of stay in the PACU (p=0.02), the number of opioid preparations (p=0.005), and the need in postoperative analgesia (p<0.05) were statistically significantly lower in EG than in CG.

**T a b l e 2:** Intraoperative characteristics and specificity of postoperative period in the studied groups, Me [Q1; Q3]

Criterion	Experimental group (n=25)	Control group (n=25)	p
Anesthesia duration (min)	165 [130; 185]	160 [125; 190]	0.27
Amount of the opiates used, 0.005% fentanyl (ml/patient)	12.0 [10.5; 15.5]	16.5 [12.0; 20.5]	0.005
OME in the intensive therapy room (per hour)	1.8 [1.1; 2.9]	3.1 [2.8; 5.2]	0.01
OME in hospital (per day)	1.1 [0.4; 1.4]	1.9 [1.1; 2.8]	0.03
Duration of stay in the PACU (h)	2 [1; 2]	5 [3; 7]	0.02
Length of hospital stay (bed-day)	7 [6; 9]	8 [6; 9]	0.06

After the operation the best MoCA and FAB indicators were found in EG compared to CG (p=0.03 and p=0.02, respectively). Impairment of memory, attention, and concentration as well as increased tiredness were registered in CG in 5 (20%) patients, none of these symptoms were noted in EG. It has been stablished that 4 patients (16%) from CG and 1 patient (4%) from EG (p=0.007) had a FAB score below 16; 3 patients (12%) from CG received a score below 26 on the MoCA, none was registered in EG. The intergroup analysis has detected mild and moderate cognitive dysfunction in 6 CG patients (24%) and in 1 EG patient (4%) (p=0.002)

**Figure 1.**
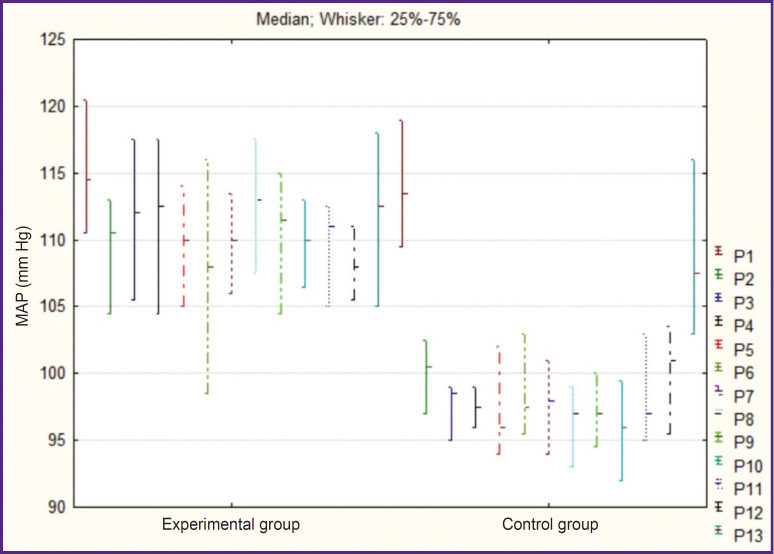
Dynamics of intraoperative indicators of mean arterial pressure (MAP) Point (P) — time indicator: before narcosis induction (P1), during skin incision (P2), further — with 25-minute intervals (up to 200 min on average, P3–P10), during operative wound suturing (P11), after extubation (P12), and 15 min after extubation (P13)

The analysis of intraoperative hemodynamics has shown significant reduction of the mean arterial pressure in CG compared to that in EG (p=0.008) and to the preoperative level (p=0.0003). In EG, no hemodynamically significant intraoperative arterial hypotension was registered (p=0.17) (Figure 1).

**Figure 2.**
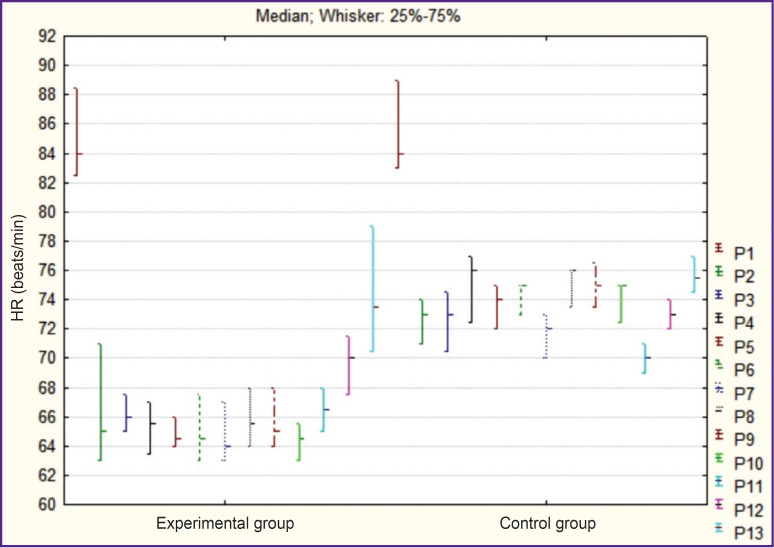
Dynamics of intraoperative indicators of heart rate (HR) Point (P) — time indicator: before narcosis induction (P1), during skin incision (P2), further — with 25-minute intervals (up to 200 min on average, P3–P10), during operative wound suturing (P11), after extubation (P12), and 15 min after extubation (P13)

The analysis of the heart rate during the operation has demonstrated insignificant bradycardia in EG (not more than 20% on average) relative to the initial level (p=0.03), while no reduction of the heart rate in CG was noted during the operation (p=0.49) (Figure 2).

**Figure 3.**
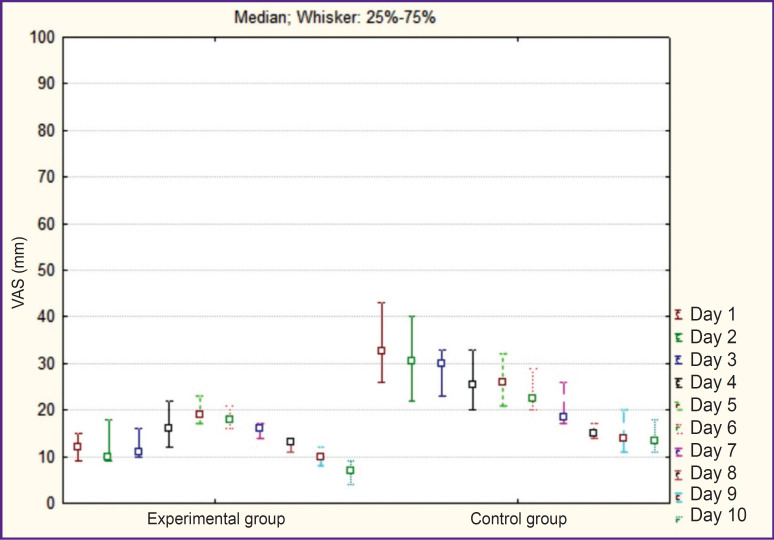
Dynamics of postoperative pain intensity on the visual analogue scale (VAS)

The level of the postoperative pain was lower in EG compared to CG during hospitalization (p<0.05) (Figure 3).

The intergroup comparison revealed fewer adverse effects of anesthesia in EG than in CG (p=0.01) at the comparable number of postoperative surgical complications (p=0.42) (Table 3).

**T a b l e 3:** Adverse effects of anesthesia and postoperative surgical complications in the studied groups

Criterion	Experimental group (n=25)	Control group (n=25)	p
***Adverse effects of anesthesia***			
Postoperative nausea and vomiting	2	4	
Bradycardia	2	1	
Dizziness	1	2	
Ineffective respiration with decreasing saturation	—	2	
Laryngospasm	—	1	
Insufficient mobility and command fulfilment	—	3	
Total, n (%)	5(20)	13(52)	0.01
***Postoperative surgical complications***			
Postoperative hematoma	1	1	
Surgical site infection	1	2	
Venous thromboembolic complications	1	1	
Total, n (%)	3(12)	4(16)	0.42

A relative risk was calculated for the total complications of anesthesia and surgery, which was equal to 0.38 (95% confidence interval (CI): 0.16–0.92) and 0.75 (95% CI: 0.19–3.01), respectively. Thus, these events in EG occurred less frequently than in the control.

## Discussion

Today, an increased attention is payed to the patients with a high neuroanesthetic risk during spinal surgical interventions [19]. It is primarily connected with a great probability of adverse effects of anesthesia, postoperative complications, and lethality as well as with a longer hospital stay and increased economic costs [20]. Overweight, elderly age, comorbid diseases, or their combinations are the main factors increasing the operational and anesthetic risk [21, 22]. Approaches based on fast-track and ERAS (enhanced recovery after surgery) technologies make it possible to perform neurosurgical interventions in this category of patients, reduce the probability of complications, and improve postoperative results [12, 23]. In the majority of cases, these approaches are non-specific and do not take into consideration the need of personified tactics.

We have previously found [24] the causes of unsatisfactory results of decompressive-stabilizing interventions at the lumbar level: female gender, age of 65 and older, body mass index of 25 or greater, ASA III and above, smoking status, blood loss exceeding 500 ml, comorbid pathology, general anesthesia longer than 180 min, a type of the surgical method. Based on the results of the mixed-effects logistic regression model, numeric values (points) were determined for each parameter and threshold values for the risk of unsatisfactory postoperative outcome: low risk up to 5 points, moderate risk from 6 to 8 points, high risk over 8 points. Based on the proposed gradation, a computer program was developed [17], which allows for a prompt assessment of the risk of unfavorable clinical outcomes in operations on the lumbar spine and determination of the appropriate tactics. The traditional anesthetic support and surgery are recommended in case of a low risk. If the risk is moderate, it is reasonable to use separate elements of our new neuroanesthetic protocol [18] at the discretion of the anesthesiologist and operating surgeon. If the risk is high, patients are strictly indicated to follow the proposed new neuroanesthetic protocol [18]. The present study is devoted to the prospective analysis of the results of using this protocol in patients with high risk of anesthesia and surgery, the assessment of its safety and effectiveness compared to the traditional anesthetic support.

At present, there are reports in the specialized literature on the complex approaches to rendering the anesthetic aid in spinal neurosurgical interventions. They are mainly aimed at: 1) minimization of tissue trauma during surgery and frequency of surgical and anesthetic complications; 2) reduction of using narcotic (opioid) preparations, shorter duration of general anesthesia and surgery time; 3) improving the safe course of anesthesia and surgery; 4) early activation and initiation of rehabilitating procedures; 5) decrease of general financial costs of postoperative treatment and rehabilitation; 6) restoration of working capacity as soon as possible [25–27]. Despite the potential advantages of the published neuroanesthetic protocols for spinal interventions, all of them do not consider preoperative stratification of surgical and anesthetic risk.

According to the data of various research groups [28–30], combinations of multimodal analgesia at different stages of neuroanesthetic support possess a high clinical effectiveness. They include antiepileptic drug (gabapentin), non-narcotic analgesics (paracetamol, Acetaminophen), nonsteroidal antiinflammatory drugs (ketorolac, ketoprofen), local anesthetics (lidocaine, bupivacaine, ropivacaine), glucocorticoids (dexamethasone, prednisolone), alpha- 2-adrenomimetics (clonidine, dexmedetomidine).

To improve the course of the perioperative period in patients with degenerative spine diseases, the following methods of anesthesia support are proposed: inhalation anesthesia [31], combined anesthesia with methadone and ketamine [32], combinations of ketamine and propofol [33], spinal anesthesia with bupivacaine [34]. A high opioid-saving effect and a small number of anesthesia-related complications are observed in the combination of minimally invasive surgical techniques with multimodal analgesia [35], intravenous sedation [36], or local anesthesia [37]. According to the systematic analysis of 31 randomized clinical studies, the reliable efficacy of the perioperative analgesia is achieved by using nonsteroidal anti-inflammatory drugs, COX-2 inhibitors, ketamine, and epidural analgesia [38], whereas an application of gabapentin and methadone is associated with a high risk of adverse drug effects and the efficacy of using local anesthetics, dexmedetomidine, glucocorticoids has not been validated [38].

The existing contradictions concerning the results of using various neuroanesthetic tactics and lack of unified algorithms of their application determine a high importance and necessity of the objectively grounded personified treatment [27]. Solution of the tasks in medicine is achieved by optimization of the results of patient treatment by processing a large set of retrospective and prospective data [39]. At the current stage of development, the choice of the tactics for rendering medical aid based on the patient dominant pathology and risk factors and prediction of unfavorable clinical outcomes are possible by the development of a CDSS [40].

The existing CDSS in treating patients with high risk of anesthesia and surgery are not numerous and in the majority of cases provide only intraoperative screening of vital functions. Besides, they do not allow surgeons to fully correct possible factors of unfavorable clinical outcome and work out a complex personified tactics [41–45].

In the present investigation, we have prospectively studied the effectiveness and safety of our new neuroanesthetic protocol [18] compared to the traditional anesthetic support in patients with a high risk of anesthesia and surgery assessed by means of the developed CDSS [17], which were operated on using the minimally invasive dorsal decompressivestabilizing technique. It has provided 1) the reduction of perioperative need in opioids; 2) stabilization of intraoperative indicators of the cardiovascular activity; 3) decrease of postoperative cognitive dysfunction frequency; 4) reduction of the local pain syndrome level; 5) fewer perioperative complications of anesthesia.

**The limitations of the study are as follows:** an open scheme and nonrandomized design; a singlecenter nature of the study; a relatively small sample; lack of investigations on the effect of symptom duration and preoperative opiate application on the postoperative pain syndrome; application of only single factor approach to the intergroup comparison using Mann–Whitney test without multiple factor analysis for the confounding control; non-inclusion of the cohort with the score below 8 on the developed CDSS into the analysis. Several components of multimodal analgesia have been examined, which did not allow us to evaluate the effect of separate preparations on the final results (however, it was included into the study design since it was our aim to determine the best scenario to minimize side-effects during the entire perioperative period). We did not compare various combinations of different components of multimodal analgesia.

## Conclusion

The developed neuroanestetic protocol of rendering specialized medical aid to the high-risk patients according to the authors’ CDSS and operated on using minimally invasive decompressive-stabilizing techniques has provided an effective postoperative analgesia and fewer drug complications.

Administration of keptoprofen before the operation, ropivacaine before the incision, dexmedetomidine intraoperatively, paracetamol during wound suturing allowed us to achieve the controlled narcosis depth without hemodynamic disorders, reduced amount of perioperatively introduced opioid drugs, fewer postoperative cognitive disturbances and complications.

Prospective randomized studies will make it possible to explore in detail the effectiveness of the proposed new neuroanesthetic protocol especially for patients with different variants of postoperative risk.
